# Research Progress of Small Molecule Fluorescent Probes for Detecting Hypochlorite

**DOI:** 10.3390/s21196326

**Published:** 2021-09-22

**Authors:** Zhi-Guo Song, Qing Yuan, Pengcheng Lv, Kun Chen

**Affiliations:** 1 The Joint Research Center of Guangzhou University and Keele Univeristy for Gene Interference and Application, School of Life Science, Guangzhou University, Guangzhou 510006, China; zhiguosong@163.com (Z.-G.S.); qingyuan2020@gmail.com (Q.Y.); 2 Zhejiang Guoneng Technology Co., Ltd., 1518 Mengxi Road, Huzhou 313000, China

**Keywords:** fluorescent probe, hypochlorite, bioimaging, sensitivity, reactive oxygen species (ROS)

## Abstract

Hypochlorous acid (HOCl) generates from the reaction between hydrogen peroxide and chloride ions via myeloperoxidase (MPO)-mediated in vivo. As very important reactive oxygen species (ROS), hypochlorous acid (HOCl)/hypochlorite (OCl^−^) play a crucial role in a variety of physiological and pathological processes. However, excessive or misplaced production of HOCl/OCl^−^ can cause variety of tissue damage and human diseases. Therefore, rapid, sensitive, and selective detection of OCl^−^ is very important. In recent years, the fluorescent probe method for detecting hypochlorous acid has been developed rapidly due to its simple operation, low toxicity, high sensitivity, and high selectivity. In this review, the progress of recently discovered fluorescent probes for the detection of hypochlorous acid was summarized with the aim to provide useful information for further design of better fluorescent probes.

## 1. Introduction

Hypochlorous acid is generally used as a bleaching agent, oxidant, and disinfectant. Industrially, HClO can be produced by, for example, the electrolysis of seawater [1]. As a strong oxidant, HClO is widely used in the disinfection of surfaces, fabrics, and other contaminated items, water, fruits and vegetables, and food and drinking utensils, cooling water treatment in coastal power stations, and bleaching agents in the paper and textile industries. In biological systems, hypochlorous acid can be regarded as the most toxic and abundant oxidant produced by white blood cells such as neutrophils and monocytes. It can quickly attack a variety of physiologically related molecules, including thiols, thioethers, amines, amino acids, nucleotides, ascorbic acid and polyenoic acid. At the same time, hypochlorous acid can generate other oxidants with very high reactivity [2,3,4,5]. Therefore, it is of great importance to realize the real-time monitoring and accurate detection of ClO^−^ in living organisms.

There are many methods that can be used to detect hypochlorous acid/hypochlorite, such as colorimetry, iodometric titration, chemiluminescence, coulometry, radiolysis, etc., [6,7,8]. However, these methods have relatively high detection lines, and the procedures are relatively cumbersome. Compared with the above-mentioned methods, the fluorescent probes have advantages such as high sensitivity, high selectivity, fast response time, and a wide detection range. Moreover, the detection process generally does not damage the sample and has little harm to cells [9,10,11,12,13,14,15,16,17]. In this paper, different categories of recently discovered ClO^−^ probes are classified based on their detection mechanisms.

## 2. Classification of Small Molecule Fluorescent Probes for Detecting Hypochlorous Acid/Hypochlorite

### 2.1. Fluorescent Probes Based on Rhodamine

The basic skeleton of rhodamine is mainly composed of two fragments of an aromatic ring connected by a carbon atom at the 9 position and a xanthene precursor with a substituted amino group at the 3 and 6 positions. Among them, the xanthene precursor with an amino group is the most important fragment because it is closely related to the fluorescence properties of rhodamine. Rhodamine compounds are fluorescent dyes based on xanthine. As its derivatives have unique spiro structure characteristics, there is no fluorescence emission. After reacting with the analyte, when the spiro ring structure is opened, the formed “open ring” form will emit strong fluorescence, accompanied by obvious color changes. The maximum emission wavelength and maximum absorption wavelength are usually above 500 nm [18]. Due to the large absorption coefficient, high fluorescence quantum yield, and excellent light stability of rhodamine-based compounds, they are usually used to design and detect metal ions, pH, anions, active oxygen, and neutral molecules with fluorescence enhancement (“turn-on”) type probe.

**Figure 1:**
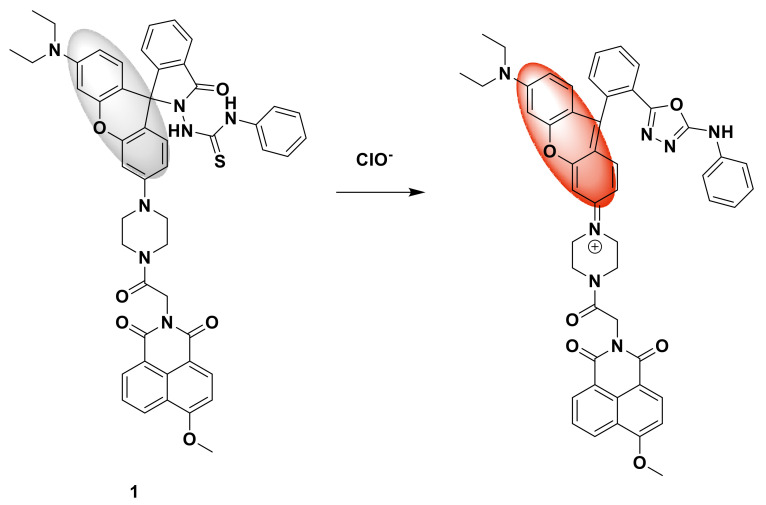
The reaction of probe 1 and ClO^−^.

Zhu et al. designed a fluorescent probe 1 based on rhodamine. The probe uses 1,8-naphthimide fluorophore as donor and rhodamine fluorophore as acceptor FRET chemical sensor [19]. Compared with a single fluorescence sensor, the ratio fluorescence chemical sensor overcomes the influence of many factors, such as the concentration of the chemical sensor, temperature, and sensitivity of the detector. It is even possible to achieve quantitative detection by self-calibrating two fluorescence emission. Under 360 nm excitation, the energy of the donor is transferred to the acceptor, resulting in a decrease in the blue fluorescence emission of the 1,8-naphthimide fluorophore at 477 nm, and the orange fluorescence of the rhodamine fluorophore at 618 nm launch enhancement (Figure 1). As hypochlorite can induce thiosemicarbazide desulfurization, promote ring-closure rhodamine to generate ring-open rhodamine, and selectively sense hypochlorite, the detection limit is as low as 4.50 nM; this fluorescent probe was successfully applied to cell staining.

**Figure 2:**
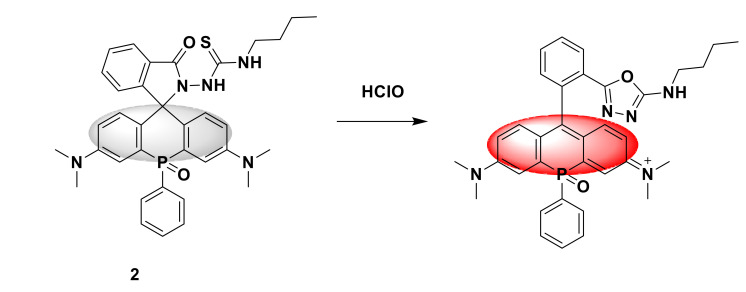
The reaction of probe 2 and HClO.

Yang and his team developed a fluorescent probe 2 (Figure 2). It is a phosphorous replacement rhodamine fluorescent probe [20]. Taking rhodamine as an example, the non-spirocyclization of the structure of rhodamine and spironolactone turns on fluorescence by manipulating the ring opening process of the analyte. Among them, thiosemicarbazide can lock the spiral ring in a non-fluorescent form, react with HClO to form oxadiazole, unlock the spiral ring, and release strong fluorescence. In addition, replacing the bridging oxygen with phosphorus atoms can produce strong fluorescence in the near-infrared region during the hypochlorous acid reaction (Figure 2), inheriting the advantages of the rhodamine structure. Fluorophores with obvious bath color transfer in the spectrum have stronger tissue penetration ability and lower phototoxicity. The designed fluorescent probe has a maximum absorption wavelength and a fluorescence wavelength of 710 nm and 730 nm, respectively. Compared with other ROS, it has high selectivity and fast response to hypochlorous acid (20 s), and the detection limit is 10 nM, benefitting from the excellent photostability, biocompatibility, solubility, and fluorescence quantum yield of the probe. Through deep imaging in vivo, fluorescent probe 2 successfully detected endogenous hypochlorite ions in mice with inflammation. In addition, the three-dimensional fluorescence imaging of endogenous hypochlorite ions in inflammatory BALB/c nude mice confirmed that the developed phosphor bridged rhodamine fluorescent probe is a promising target for generalization in biological systems point depth imaging bracket.

**Figure 3:**
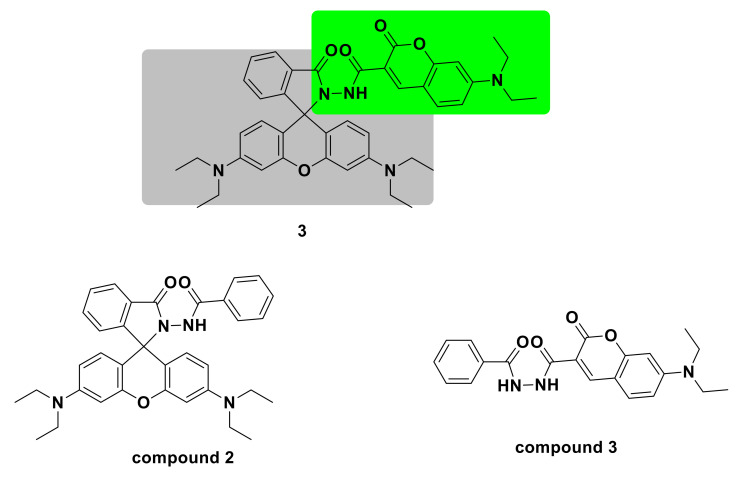
The structure of probe 3.

**Figure 4:**
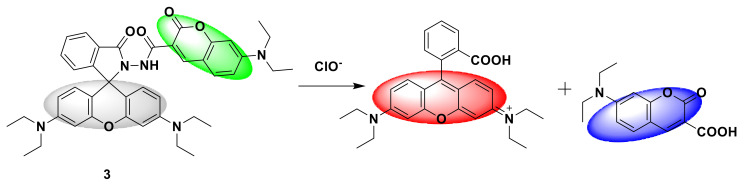
The reaction of probe 3 and ClO^−^.

Long et al. [21] designed a new type of dual fluorophore probe 3. The fluorescent probe is composed of compound 2 containing rhodamine fluorophore and compound 3 containing coumarin fluorophore (Figure 3). Although the previous dual-fluorophore probes responded after the ClO^−^ reaction, the emission peaks of the two fluorophores overlapped severely, and accurate detection was not possible. The advantage of the new fluorescent probe is that after reacting with 80 μM ClO^−^, the two fluorophores of the probe respond simultaneously in two independent optical windows, which greatly improves the sensitivity. The fluorescence ratio (I_578_/I_501_) increased from 0.01 to 39.55, which was a 3955-fold increase. It shows a good linear relationship with ClO^−^ in the range of 0.2–40 μM, and the detection limit is 0.024 μM. The response mechanism is that the fluorescence of the two fluorophores changes to produce two new fluorescent molecules (Figure 4). The fluorescent probe has been successfully applied to the detection of ClO^−^ in natural water.

**Figure 5:**
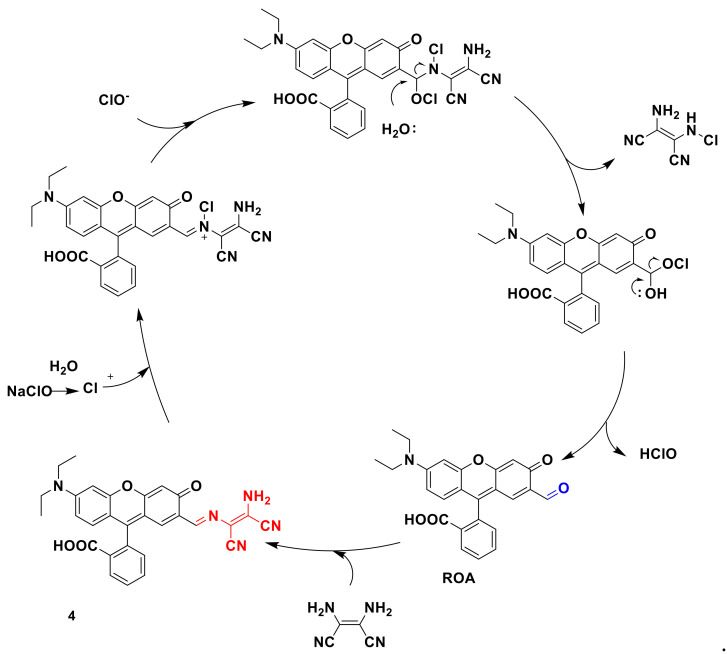
The recognition mechanism of probe 4.

A new “turn on” fluorescent probe 4 [22] was synthesized by Zhang and his research team (Figure 5), which, based on rhodamine fluorophore, can be used to detect ClO^−^. In this paper, a new red pigment, ROA (20-formyl red pigment), is designed by the condensation reaction of ROA’s formyl group as a fluorophore with diaminomaleonitrile to form a fluorescent probe 4. The formation of the formyl group caused by the oxidative hydrolysis of C=N makes the probe almost non-fluorescent. After ClO^−^ is added, it shows strong yellow fluorescence. The recognition mechanism is shown in Figure 5. The new fluorescent probe exhibits a stable fluorescent signal between pH 5 and 9, and shows high selectivity to ClO^−^, high extinction coefficient, and short-term response. The imaging studies of A549 cells and live mice show that probe 4 can be used as an effective fluorescent probe for detecting ClO^−^ in biological systems.

**Figure 6:**
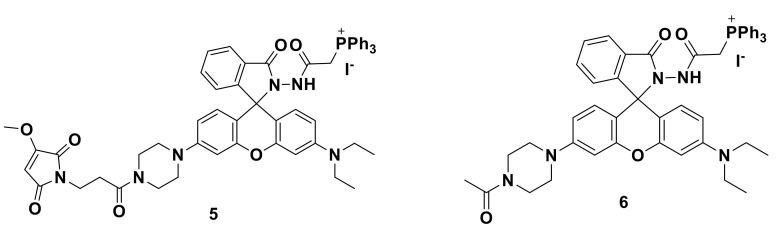
The structures of probe 5 and probe 6.

**Figure 7:**
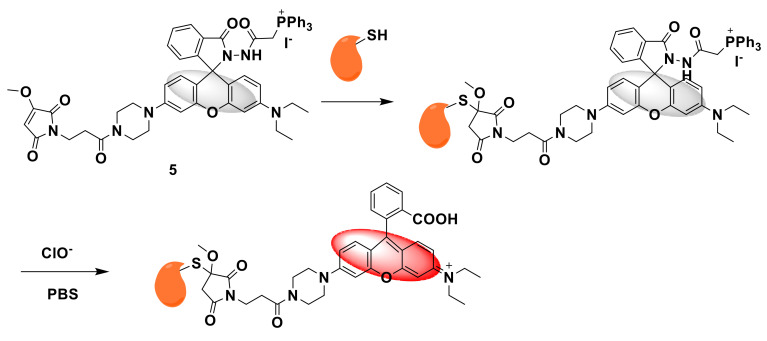
The reaction of probe 5 and ClO^−^.

Li’s team developed a mitochondrial immobilized fluorescent probe 5 and 6 for selective detection of hypochlorite [23]. As shown in Figure 6, probe 5 uses a benzoyl acetyl hydrazine linker to connect triphenyl phosphine (TPP) to the rhodamine fluorophore, where TPP has mitochondrial targeting and benzoyl hydrazide is ClO^−^ as the reaction site, triphenylphosphine is the target, and the methoxymaleimide immobilization group is used as the immobilization site to react with nucleophilic reagents such as mitochondrial peptides to form a covalent bond to fix the probe in the mitochondria. For comparison, probe 5 has no fixed site. Studies have found that the reactivity of methoxymaleimide with Cys is better than that of GSH and can be stored for a long time in water. The optical properties show that the two probes have almost no fluorescence without ClO^−^. With the addition of ClO^−^, the fluorescence intensity at 585 nm gradually increases at the excitation wavelength of 530 nm, and the color changes to red. The response mechanism of probe 5 is shown in Figure 7. Fluorescence imaging showed that after different stimuli caused the mitochondrial membrane potential (MMP) to decrease, the retention of probe 5 in the mitochondria was enhanced, and the imaging performance was better than probe 6, indicating the advantages of this design strategy. Finally, probe 5 was used to detect endogenous ClO^−^ in tissues and zebrafish.

### 2.2. Fluorescent Probes Based on BODIPY

As an excellent fluorophore, boron dipyrromethene (BODIPY) has been introduced into HClO probes in recent years [24]. It has many advantages, such as high light stability, high molar absorption coefficient, narrow absorption and emission peaks, high fluorescence quantum yield, easy functionalization, etc. By changing the conjugation and substituents and modifying the structure, the emission and absorption wavelengths can be changed to the near-infrared light region or the ultraviolet-visible light region to achieve the purpose of detection. However, BODIPY derivatives also have disadvantages such as poor water solubility, insensitivity to pH and small Stokes shift, so the development of BODIPY probes still needs further research [25,26].

**Figure 8:**
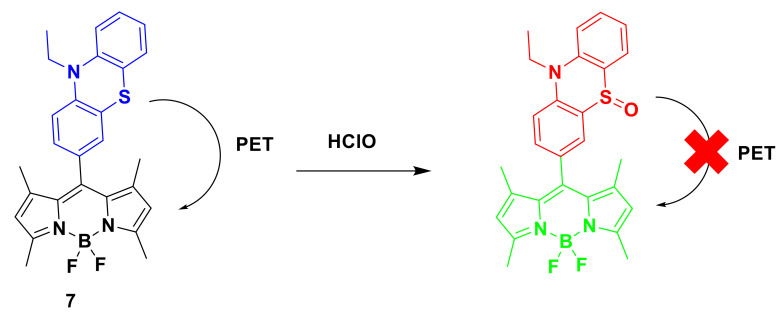
The reaction of probe 7 and HClO.

For instance, Li et al. designed and synthesized a new type of bodipy-based fluorescent probe 7 (Figure 8), which can detect ClO^−^ sensitively and selectively [27]. The structure of probe 7 was clearly characterized by ^1^H NMR, ^13^C NMR, and ESI-MS spectra. Due to the PET effect of the phenothiazine part, the emission of the probe is essentially quenched. After the reaction with ClO^−^, the phenothiazine group is oxidized to its sulfoxide form, so that the PET process is prevented and BODIPY is restored. Strong green light was emitted from the unit. Compared with other ROS, probe 7 shows excellent selectivity to ClO^−^. The detection limit is as low as 1.7 nM, which proves the sensitivity of the probe. The fluorescence response of probe 7 (1μM) to ClO^−^ was investigated in PBS buffer (containing 50% EtOH as a cosolvent) at room temperature. With the continuous increase of ClO^−^ concentration, the fluorescence intensity of the probe at 515 nm gradually increased, resulting in an OFF–ON type fluorescence response to ClO^−^. At the same time, the fluorescent probe showed a fast response in just a few seconds. Moreover, the new probe has been proved to be effective in visualizing changes in exogenous ClO^−^ in live HeLa cells. Co-location studies using Mito Tracker red show that the probe can track changes in ClO^−^ content in mitochondria.

**Figure 9:**
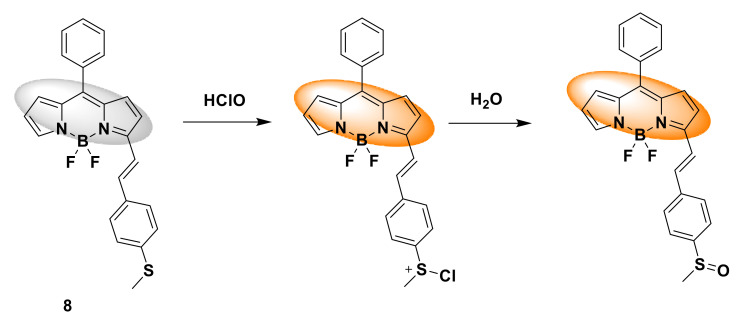
The reaction of probe 8 and HClO.

Chu et al. developed a new fluorescent probe 8, consisting of BODIPY fluorophore and methyl phenyl sulfide [28]. When the quantum yield is 0.006, due to the photo-induced electron transfer (PET) of methyl phenyl sulfide to the fluorophore BODIPY, the fluorescence emission of the probe is negligible. After the reaction with HOCl, the oxidation of methyl phenyl sulfide to methyl phenyl sulfoxide hinders the PET process and further restores the strong orange-yellow fluorescence emission of BODIPY. The response mechanism is that HClO first generates unstable sulfinyl chloride intermediates through chlorination on sulfur, and then reacts with water to form sulfoxide compounds (Figure 9). The time response is under study. The fluorescence emission of the probe reaches its maximum intensity within 30 s, indicating that this is a fast response. In addition, the titration data showed that in an aqueous methanol solution (*v*/*v* = 7/3, 0.1 M PBS, pH 7.4) with an excitation wavelength of 530 nm, probe 8 (5 μM) exhibited a maximum emission intensity at 579 nm. At a concentration of 0–10 μM HClO, the titration curve shows a linear relationship. The detection limit is 30 nM. In the selectivity evaluation, strong orange-yellow fluorescence is emitted only in the presence of HClO, indicating high selectivity. In cell imaging studies, RAW264.7 cells were used to detect the response ability of fluorescent probe 8 to HClO. After adding the probe, RAW264.7 cells did not show any fluorescence, indicating that the intracellular HClO content was low. After adding NaOCl, the cells showed strong yellow fluorescence, indicating that the probe has high specificity for detecting endogenous HClO levels in living cells.

**Figure 10:**
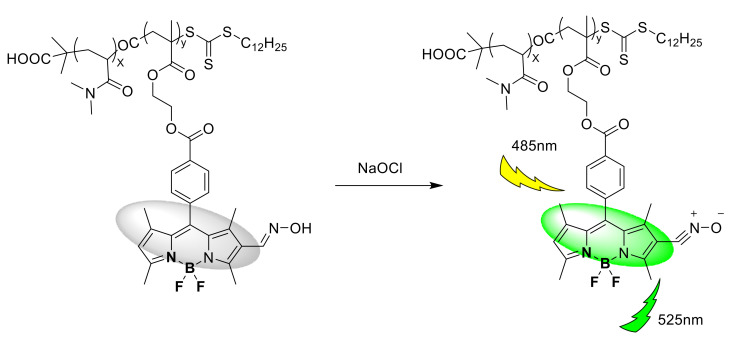
The reaction of probe 9 and ClO^−^.

In addition, Halder and co-workers developed a boron dipyrromethene (BODIPY)-based polymer probe 9 (Figure 10) for the detection of HClO in pure water (pH = 7.4) [29]. Under 485 nm excitation, with the gradual addition of NaOCl (0–1.0 μM), the emission intensity of the probe (2.5 × 10^−4^ M) at 525 nm increased sharply, and the fluorescence quantum yield (Φ) increased from 0.04 to 0.34, the fluorescence intensity has a good linear correlation (R^2^ = 0.988), and the detection limit is as low as 17 nM. The response time is short, reaching a plateau within 49 s. The probe solution of 2.5 × 10^−4^ M was stored at room temperature for 7 days, and the fluorescence intensity hardly changed to verify its photolysis stability. The MTT method was used to detect the toxicity of the probe to HepG2 cells and W138 cells. When the probe concentration is 200 μg/mL, the cell viability can reach 80–82%, indicating that the probe is less toxic. After incubating with the probe (10 μM) for 20 min, it showed weak fluorescence. After adding sodium hypochlorite (10 μM) and incubating for 10 min, the cells showed obvious green fluorescence, which confirmed that the probe has stable fluorescence characteristics in biological systems. In vitro imaging of exogenous HClO in live HepG2 cells.

**Figure 11:**
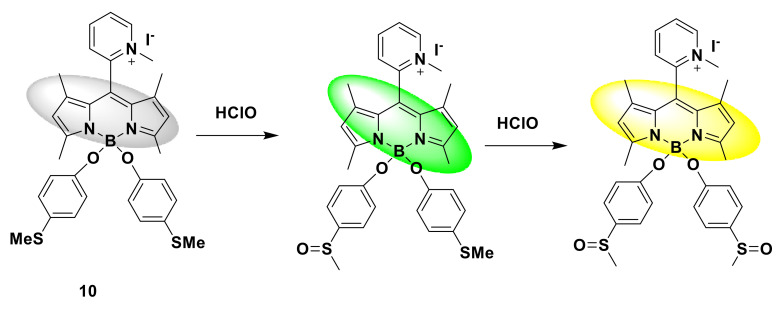
The reaction of probe 10 and HClO.

Jin et al. designed a water-soluble fluorescent probe 10 based on BODIPY [30]. The first is to modify the BODIPY structure. Two methyl mercaptophenoxy groups, respectively, replace fluorine atoms to induce light-induced electron transfer to quench BODIPY’s fluorescence. The probe has extremely low fluorescence (Φ = 0.0013) in PBS (10 mM, pH 7.4). After adding HClO, the probe produced a significant response within 1 min, and a significant fluorescence enhancement of 100 times. At 0–10 μM HClO concentration, the titration curve shows a linear relationship. The detection limit is 53 nM. The response mechanism of the fluorescent probe is that the two methyl mercaptophenoxy groups are oxidized by HClO and cause the fluorescence to “turn on” (Figure 11). Cell imaging experiments show that the probe has been successfully applied to the imaging of live cell HClO.

**Figure 12:**
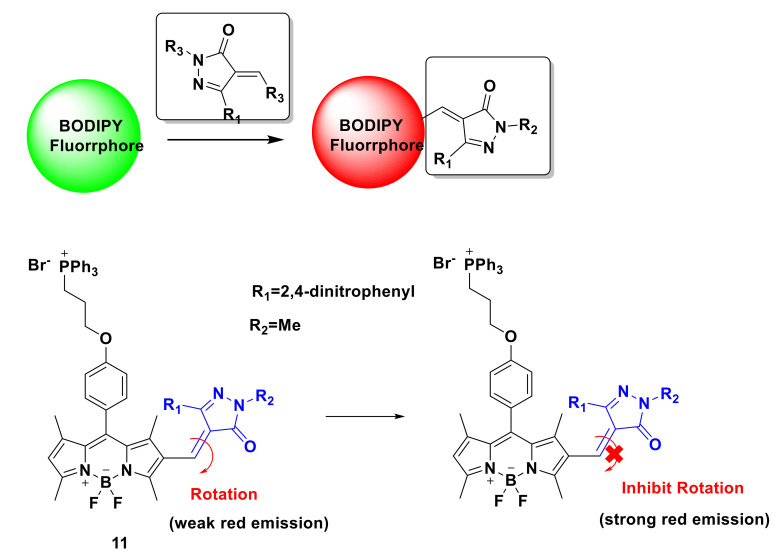
The structure of probe 11.

**Figure 13:**
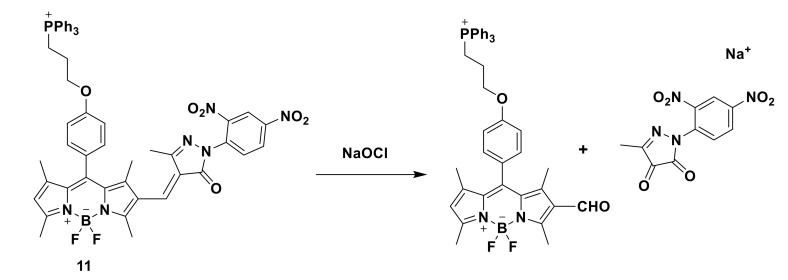
The reaction of probe 11 and ClO^−^.

Xu’s team used the α,β-unsaturated pyrazolone backbone and BODIPY fluorophore to prepare a new fluorescent probe 11 [31]. In this paper, 3-methyl-(2,4-dinitrobenzene)-2-pyrazolin-5-one is selected as the model of α, β—unsaturated pyrazolone. The probe with C=C double bond has weak red fluorescence, but the limitation of TICT promotes the enhancement of red fluorescence. In this paper, 3-methyl-1-(2,4-dinitrobenzene)-2-pyrazolin-5-one is selected as the model of α, β—unsaturated pyrazolone. The probe with C=C double bond has weak red fluorescence, but the limitation of TICT promotes the enhancement of red fluorescence (Figure 12). In the study of optical performance, the new probe has high selectivity, high sensitivity, and suitable pH range. The sensing mechanism of the fluorescent probe to detect hypochlorite is also verified by MS spectrum analysis, ultraviolet–visible spectrum and emission spectrum. Under the action of NaOCl, the probe reacts with ClO^−^ to generate the corresponding aldehyde group (Figure 13). In the fluorescence imaging experiment, MCF-7 cells were stained with a 10 μM probe, and weak fluorescence was observed after 15 min. After incubation with 10 μM NaOCl for 10 min, the fluorescence increased significantly. The results showed that the probe could be used to detect hypochlorite in living cells. Then, the zebrafish fluorescence imaging experiment was carried out. After incubation for 45 min with the probe (10 μM), zebrafish showed weak red fluorescence and strong green fluorescence. The results showed that the probe was sensitive to hypochlorite in vivo. Further development is needed to screen chemical catalysts and prodrugs by fluorescence analysis.

**Figure 14:**
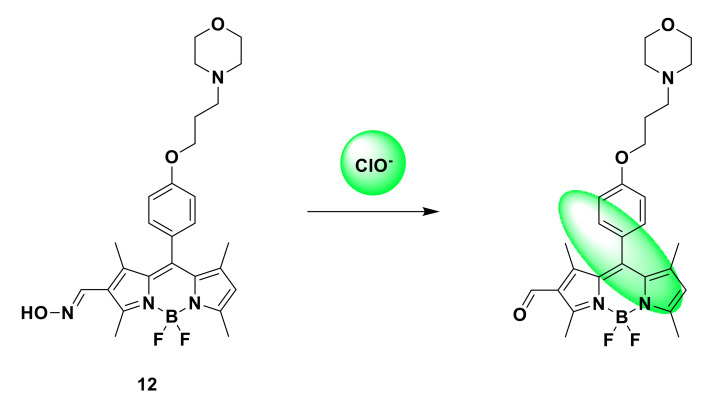
The reaction of probe 12 and ClO^−^.

According to the similar sensing mechanism of probe 9, Huang et al. synthesized a fast and sensitive fluorescent probe 12 based on the BODIPY group [32]. The combination of BODIPY fluorescence and aldoxime unit can selectively detect ClO^−^ within 60 s. Due to the C=N isomerization mechanism of BODIPY at position 2, extremely weak fluorescence is produced. After adding HClO, the fluorescence emission of the probe was turned on, the fluorescence intensity of the probe was significantly increased by 29 times at 530 nm, and the quantum yield increased from 0.03 to 0.25 (Figure 14). It has also been successfully used to monitor endogenous and exogenous hypochlorous acid in MCF-7 cells. Fluorescence imaging shows that the probe can locate lysosomes in living cells.

### 2.3. Fluorescent Probes Based on Fluorescein

Fluorescein is also known as fluorescent yellow. Fluorescein is the matrix of luminescent materials. As the oxygen bridge bond fixes the two benzene rings on the same plane, the molecule has a rigid coplanar structure, which is conducive to the generation of fluorescence. Therefore, it is widely used. Fluorescein probes have good properties, such as high quantum yield, easy synthesis [33,34,35], and good biocompatibility [36]. It has become a research hotspot in recent years.

**Figure 15:**
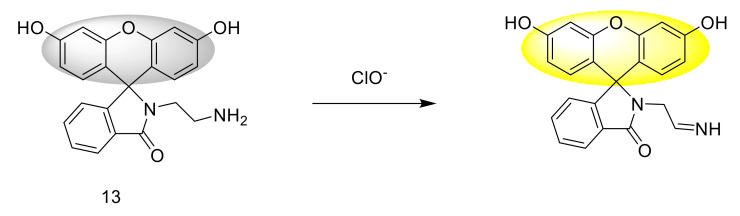
The reaction of probe 13 and ClO^−^.

A fluorescent probe 13 based on fluorescein was designed and synthesized [37], which is a “turn-on” type probe. It has high sensitivity, high selectivity, and a fast response time. The response mechanism is that after reacting with ClO^−^, hypochlorite oxidizes the amino group in the probe to imine, making the colorless solution of the probe turn pink (Figure 15). The response mechanism is also carried out by ESI-MS and -^1^H NMR verification. The results show that hypochlorite can oxidize the amino group in the probe structure. Under the excitation of 490 nm, the fluorescence increase at 553 nm is 150 times. The maximum absorption band is at 479 nm. The detection limit is as low as 0.023 mM. Through fluorescence confocal imaging experiments, the results show that the probe can be used for the detection of ClO^−^ in HeLa cells.

**Figure 16:**
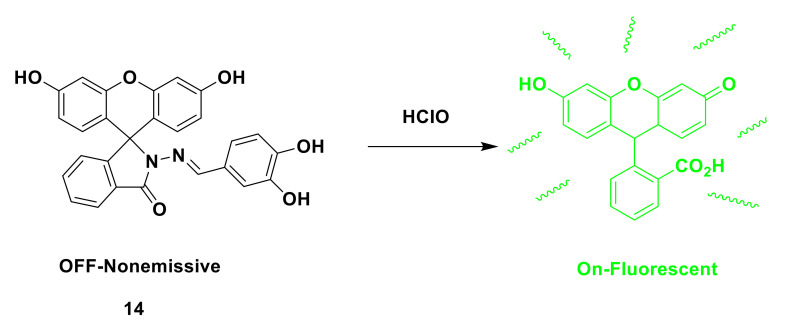
The reaction of probe 14 and HClO.

**Figure 17:**
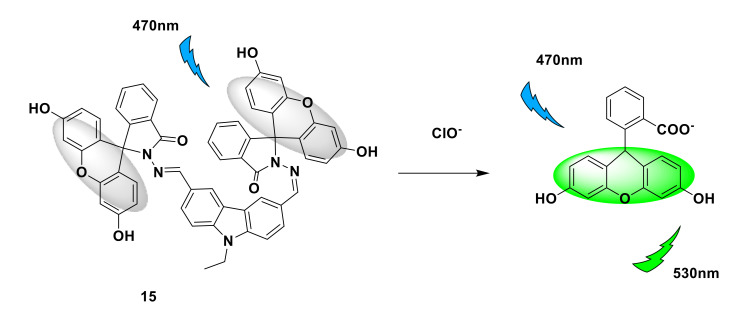
The reaction of probe 15 and ClO^−^.

Similar to the above strategy, Yin et al. also designed and synthesized a “turn-on” probe 14 based on the fluorescein structure [38]. The closed structure of the spironolactam in the probe leads to no light. After the HClO reaction, the catechol is oxidized, and the ring of the spironolactam turns on strong green fluorescence (Figure 16). In Tris-HCl buffer solution (pH = 7.4), the sensitivity of the probe to ClO^−^ was evaluated and fluorescence titration was performed. After adding NaOCl (aq), the emission wavelength appeared at 523 nm under the excitation of 490 nm. After adding 15 equivalents of ClO^−^, the emission intensity reached the maximum. The fluorescence quantum yield is Φ = 0.87, which is 1240 times higher than its own. Fluorescence imaging experiments show that the probe can be used for the detection of endogenous and exogenous hypochlorous acid in living cells. The probe may be a useful tool to study the role of HClO in pathological processes. Similar to the response mechanism of probe 14, Wang et al. [39] designed and synthesized a fluorescent probe 15 with fluorescein-carbazole as the backbone. After reacting with ClO^−^, the C=N bond is broken, the closed structure of spirolactam is opened, and the fluorescence is enhanced (Figure 17). Fluorescence imaging experiments also verified that the probe can be used for the detection of ClO^−^ in living cells and zebrafish.

**Figure 18:**
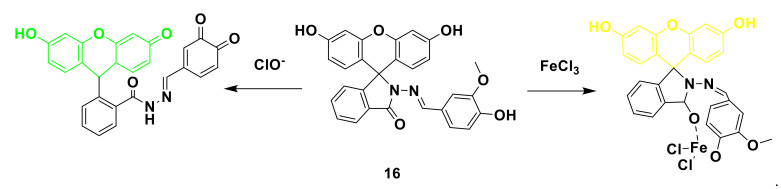
The reaction of probe 16 and ClO^−^.

Jin’s group [40] present a novel fluorescein-derived dual function probe 16. The probe can detect Fe^3+^ by naked eyes and ClO^−^ for fluorescence detection. The spirolactam ring structure of the fluorescein derivative in the probe itself does not emit light, and only shows weak fluorescence in methanol-PBS (5/5, *v*/*v*, pH 7.4) buffer solution. After interacting with ClO^−^, it emits green fluorescence (Figure 18). When 3.5 equivalents of ClO^−^ are added, it reaches saturation, the fluorescence intensity is increased by 102 times, and the quantum yield is 0.56. It shows that the probe can perform quantitative and qualitative detection of ClO^−^. After the probe interacts with Fe^3+^, the absorption peak at 342 nm gradually increases, and the color of the probe solution can be observed with the naked eye from colorless to yellow. It shows a linear relationship in the range of 2–30 μM, and the detection limit is 100 nM. Adding ClO^−^, other anions, and metal ions to the probe solution, it was found that there was only a response to ClO^−^. In the application experiment, the probe can detect ClO^−^ and Fe^3+^ in the tap water on the campus of Xi’an Technological University, providing a potential tool for future water sample detection.

**Figure 19:**
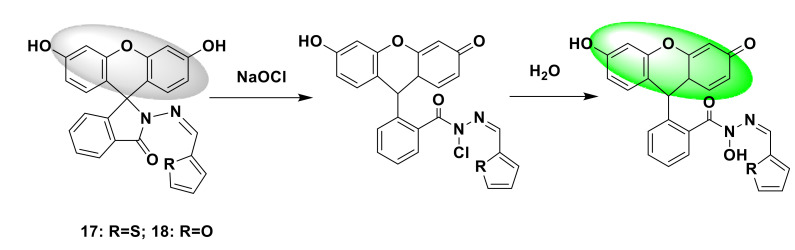
The reactions of probes 17, 18 and ClO^−^.

In addition, Jin et al. proposed two new types of fluorescent chemical sensors 17,18 based on fluorescein [41]. With the introduction of S and O heterocycles based on the modification of the fluorescein structure (Figure 19), the response mechanism is similar to the above fluorescein derivative structure probe; adding ClO^−^ causes the opening of the spirolactam ring and emits a strong fluorescent signal. Probe 17 showed higher sensitivity, so further research was conducted with it. The product after the response of probe 17 was structurally characterized by an ESI-MS spectrometer, and a new peak appeared as a product peak. The optical performance test shows that the probe has a short response time, high sensitivity, high selectivity, and stable pH application range. In practical applications, the probe is used to detect different water samples. After ClO^−^ is not added, there is no fluorescence response. After the addition, the fluorescence response is generated, and it increases with the increase of the added amount. A quantitative analysis of ClO^−^ was carried out. Then, to verify the application in biology, Rhodobacter ferrooxidans sp. was chosen. SW2 cells were used as the research model. In low-phosphate freshwater mineral medium [42,43], probe 17 was incubated with the cells at 20 °C for 1 h, and no fluorescence signal was detected. After 70 µM ClO^−^, incubated for another 20 min, the fluorescence intensity increased by more than 20 times, indicating that the probe can be used for the detection of ClO^−^ in biological samples.

### 2.4. Fluorescent Probes Based on Coumarin

Coumarin has attracted the attention of scientific researchers because of its low price, low toxicity, easy modification, large Stokes shift, and excellent fluorescence performance. Some fluorescent probes based on coumarin groups have been designed and synthesized for ClO^−^ [44,45,46]. However, there are some disadvantages, such as long reaction process and poor solubility in water. Therefore, it is still necessary to develop novel fluorescent probes based on coumarin groups for production and daily applications.

**Figure 20:**
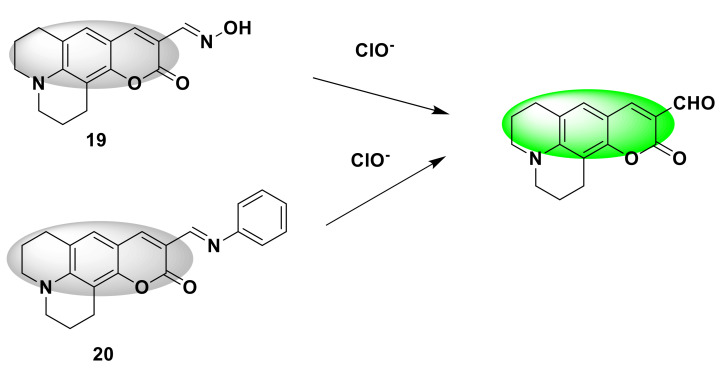
The reactions of probes 19, 20 and ClO^−^.

For example, Cheng et al. designed and synthesized two novel coumarin-type fluorescent probes 19 and 20 [47]. The coumarin and oxime protecting group form the recognition group, which is a fluorescence turn-on probe. After ClO^−^ is added, the imine group is converted into an aldehyde group. Under the irradiation of ordinary ultraviolet lamps, the solution changes from no fluorescence signal to strong green fluorescence (Figure 20). Whilst increasing the ClO^−^ (2 µmol/L) concentration, the fluorescence intensity of probe 20 at 510 nm increased by about 85 times, while the fluorescence intensity of probe 19 only increased by 1.28 times, and the sensitivity of probe 20 was higher. Considering the practical application, probe 20 is used for further discussion. When evaluating selectivity, all other anions have almost no obvious changes in fluorescence, but only show high selectivity to ClO^−^. The results of bioimaging experiments are consistent with the results of titration experiments. Probe 20 has good cell membrane penetration and has the potential to be used for ClO^−^ imaging in live cells.

**Figure 21:**
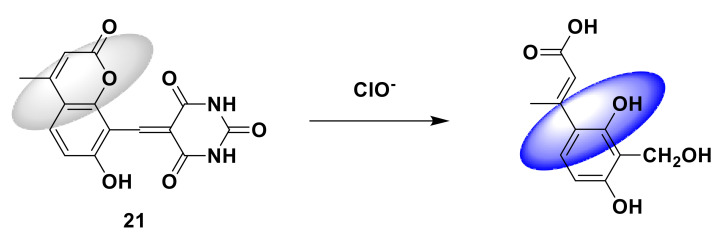
The reaction of probe 21 and ClO^−^.

Xie et al. [48] proposed a simple fluorescence sensor 21 (Figure 21) based on coumarin derivatives, which is obtained by the condensation reaction of barbiturates and coumarin derivatives. When a series of concentrations of ClO^−^ (0–50 μM) titrate the probe solution, as the concentration increases, the UV absorption bands at 259 nm and 325 nm gradually decrease, while the UV absorption bands at 385 nm and 480 nm gradually increase. The solution turns colorless to orange. At an excitation wavelength of 385 nm, the fluorescence emission at 500 nm is blue-shifted to 458 nm. The fluorescence quantum yield increased from 0.058 to 0.526. The detection limit is 0.73 μM. When the pH is 3–11, the fluorescence intensity remains stable. The probe can easily detect ClO^−^ in the water sample, and the test paper with the probe is prepared, and there is almost no fluorescence on the test paper under 365 nm ultraviolet rays. When ClO^−^ solutions of different concentrations were dropped onto the test paper, the fluorescent color of the test paper slowly changed from colorless to blue.

**Figure 22:**
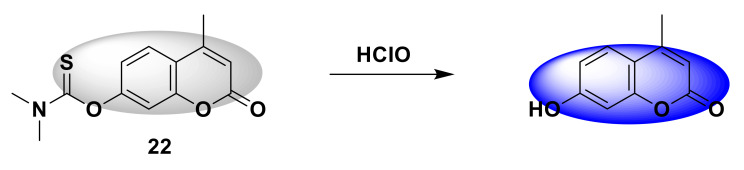
The reaction of probe 22 and HClO.

**Figure 23:**
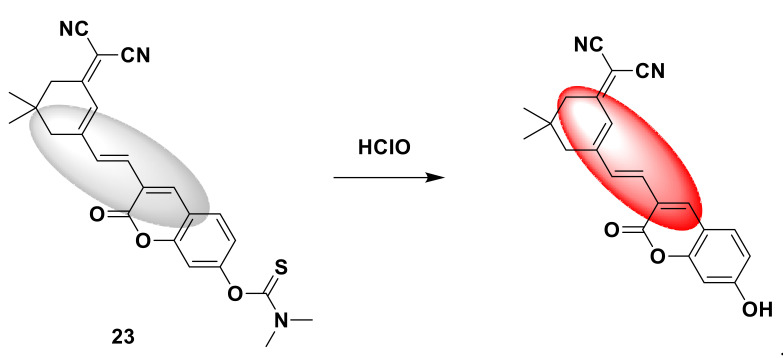
The reaction of probe 23 and HClO.

**Figure 24:**
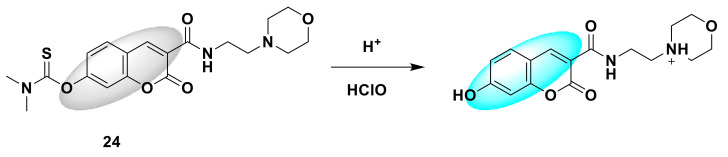
The reaction of probe 24 and HClO.

Wang and his colleagues developed an open-type fluorescent probe 22 [49]. The synthesis method is simple and requires only one step. It is prepared by reacting 4-methylumbelliferone (coumarin derivative) with dimethylcarbamoyl chloride. Dimethylcarbamethanesulfonyl chloride is the reaction site. When encountering HClO, the sulfide part will be oxidized, releasing coumarin derivatives and showing obvious fluorescence (Figure 22). The response time is short, reaching the maximum within 60 s, and then tends to stabilize, and the detection limit drops to 34.75 nM. In addition, the probe is also used to detect HClO in tap water and living cells. It provides a potential tool for the detection of HClO in environmental systems and biological systems in the future. In addition, Zhang and his colleagues synthesized a new near-infrared fluorescent probe 23 [50] with large Stokes shift, with coumarin-dicyanoisophorone as the fluorescence response group, and dimethyl thioaminomethyl acid ester response site. It is a turn-on probe. There is no obvious fluorescence. After adding ClO^−^, there is obvious red fluorescence at 700 nm (Figure 23), the fluorescence is enhanced by more than 200 times, and the Stokes shift is larger (190 nm). The advantages of this probe are its short response time (<2 s) and high selectivity. Biological imaging experiments verified that fluorescent probes can be effectively applied to the ClO^−^ detection of living cells, zebrafish, and mice. All results show that the probe is a potential near-infrared fluorescence imaging tool. Similar to the above-mentioned probe design strategy, Shi et al. designed a fluorescent sensor 24 [51] that can target lysosomes with *N,N*-dimethylthiocarbamate (DMTC) as the reaction site. The legume fluorophore is the response group. Since the morpholine group and the hydroxyl group are pH-sensitive parts, the synthesized probe will only respond in an acidic environment. After adding HClO, *N,N*-dimethylthiocarbamate breaks away, the fluorescence is turned on, and blue fluorescence is generated (Figure 24). Quantitative detection can be achieved when the range of HClO is 0–80 mmol/L, and the detection limit is as low as 24.3 nmol/L. It can detect exogenous and endogenous HClO in lysosomes of living cells.

**Figure 25:**
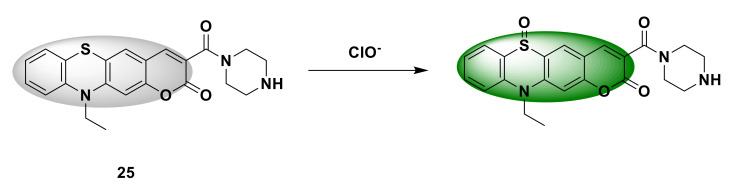
The reaction of probe 25 and ClO^−^.

Hou et al. [51] developed a new probe 25 (Figure 25) for detecting ClO^−^, which combines coumarin with benzothiazine and adds a piperazine unit to improve cell membrane permeability and solubility. The sulfur atom in the probe can be oxidized by ClO^−^ and emit green fluorescence. When ClO^−^ (0–200 μM) was added to the probe solution, the fluorescence intensity of the probe at 520 nm gradually increased and reached saturation when 140 μM ClO^−^ was added. When the concentration of ClO^−^ is between 0 and 10 μM, the fluorescence intensity of the probe increases linearly. MTT experiment confirmed that the probe has low toxicity. After incubating MCF-7 cells with the probe (10 μM) for 30 min, there was almost no fluorescence in the cells. After adding 50 μM NaOCl, green fluorescence was produced. Pretreating MCF-7 cells with IFN-γ (50 ng/mL) and LPS (1μg/mL) for 24 h, and then incubating with the probe for 30 min, can also produce green fluorescence, indicating that the fluorescent probe can be used for living detection of endogenous and exogenous ClO^−^ in cells.

### 2.5. Fluorescent Probes Based on 1,8-Naphthalimide

1,8-Naphthimide is a common fluorophore in the design of fluorescent probes. It has the characteristics of high fluorescence quantum yield, large Stokes shift, stable optical performance, and good biocompatibility [52,53,54,55,56,57]. Applications in the field of biological fluorescent probes include pH probes [58,59,60], ionic probes [61,62,63,64], viscosity probes [65], small molecules and macromolecular [66,67,68,69,70,71,72,73] probes, and so on. In recent years, the use of fluorescent probes based on 1,8-naphthimide derivatives to detect hypochlorous acid in biological systems has attracted more and more attention from researchers.

**Figure 26:**
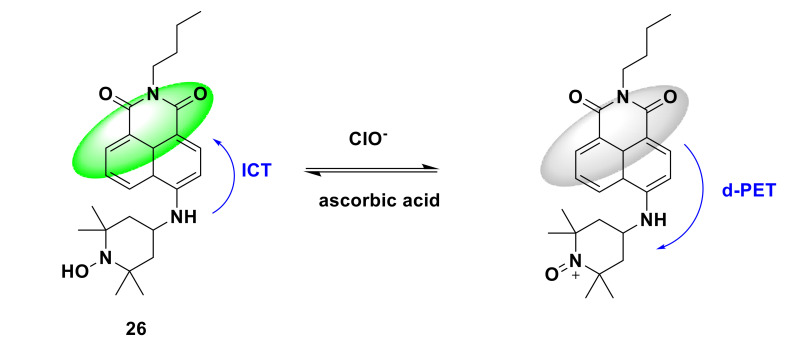
The reaction of probe 26 and ClO^−^.

He et al. developed a naphthalimidebased reversible fluorescent probe 26 [74]. The hydroxylamine in the probe can be oxidized to ammonium oxide by HClO/ClO^−^, and then the ammonium oxide is reduced to hydroxylamine by ascorbic acid. Under the excitation of 475 nm, bright yellow-green fluorescence emission was emitted into the 10 μM probe solution. After adding 20 μM ClO^−^ and standing for 5 min, the fluorescence was quenched (Figure 26), and the fluorescence quantum yield also decreased from 20.58% to 0.07%. The response is extremely fast, stable within 8 s. Thirty minutes after adding ascorbic acid, the fluorescence gradually returned to its original brightness. After four reversible cycles, there was only a moderate decrease in fluorescence intensity, which indicated that the probe could be regenerated. Common anions, reducing amino acids, reactive oxygen species and ClO^−^ are added to the probe solution. Only after adding ClO^−^, the fluorescence is almost completely quenched, and there is almost no such result for other substances, which indicates that the probe has good selectivity to ClO^−^. In addition, the designed novel probe can be applied to the detection of endogenous and exogenous ClO^−^ in living cells.

**Figure 27:**
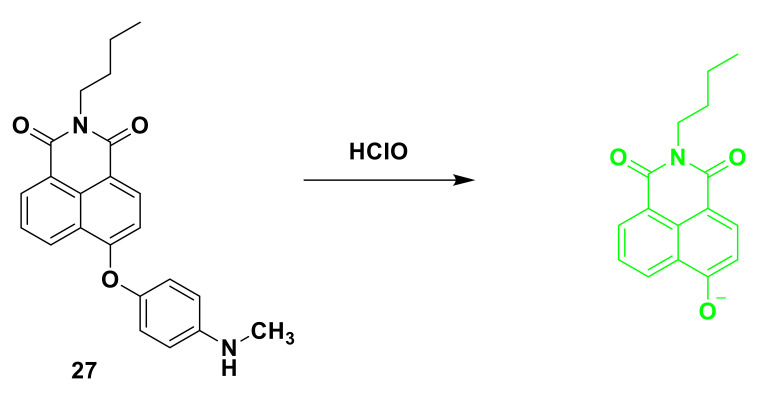
The reaction of probe 27 and HClO.

A new fluorescent probe 27 was synthesized by 4-hydroxy-1,8-naphthimide and *p*-methylaminophenyl ether. The advantage of this probe (Figure 27), designed by Wang et al. [75], is that 4-hydroxy-1, 8-naphthalimide is an excellent fluorophore, and p-methylaminophenyl ether is used as a recognition group. When the probe encounters hypochlorous acid, it emits a strong fluorescence response. There is a main absorption peak at 372 nm in the absorption spectrum of the probe. When hypochlorous acid was added to the probe solution, a new absorption peak appeared at 465 nm. The red-shifted absorption spectrum proves the creation of a new structure. It is characterized by its structure as 4-hydroxy-1,8-naphthimide. The reaction mechanism between the probe and hypochlorous acid 1 was determined, and the methylamino phenyl ether part of the probe HOCl was oxidized and separated. In water and ethanol buffer solution (10% ethanol, 10 mM PBS, pH = 7.4), the fluorescence intensity of the probe at 550 nm is negligible. After hypochlorous acid is added, the fluorescence response intensity reaches the maximum in about 1 min value. The probe has a good linear relationship with (0–1 μM) hypochlorous acid, and the detection limit is as low as 6.56 nM. The probe can selectively detect hypochlorous acid instead of common anions, metal ions, mercaptans, and other reactive oxygen species. In addition, the probe can detect endogenous/exogenous hypochlorous acid in live cells and zebrafish.

**Figure 28:**
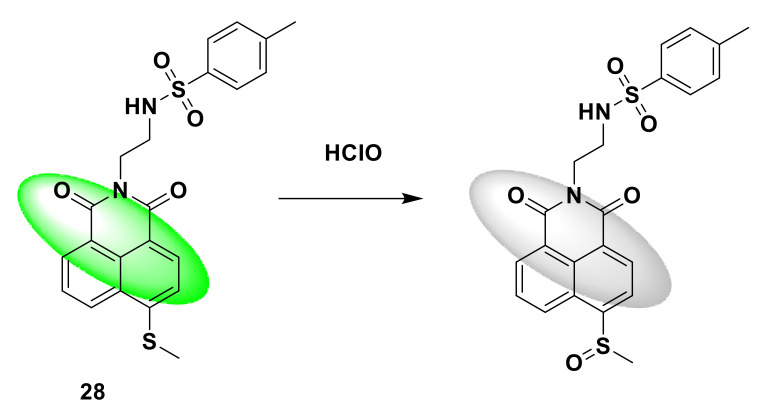
The reaction of probe 28 and HClO.

Recent studies have shown that a lack of sleep is closely related to ROS in the body, which can induce endoplasmic reticulum stress. Therefore, Xia et al. developed a naphthimide-based two-photon fluorescent probe 28 with endoplasmic reticulum targeting to monitor ROS generated during sleep deprivation [76]. The probe has a fast response (about 150 s) and high sensitivity to HClO. The probe itself emits green fluorescence. After reacting with HClO, the sulfur atom connected to the naphthimide is oxidized, which changes the fluorescence effect and produces white fluorescence (Figure 28), ^1^HNMR, ^13^C NMR, and other structural characterizations. By taking the fluorescence confocal shooting of HeLa cells and zebrafish, it can be concluded that this new probe can be used to monitor the production of endogenous and exogenous hypochlorous acid in living cells and zebrafish. It is also the first discovery that sleep insufficiency can induce hypochlorous acid production in zebrafish.

**Figure 29:**
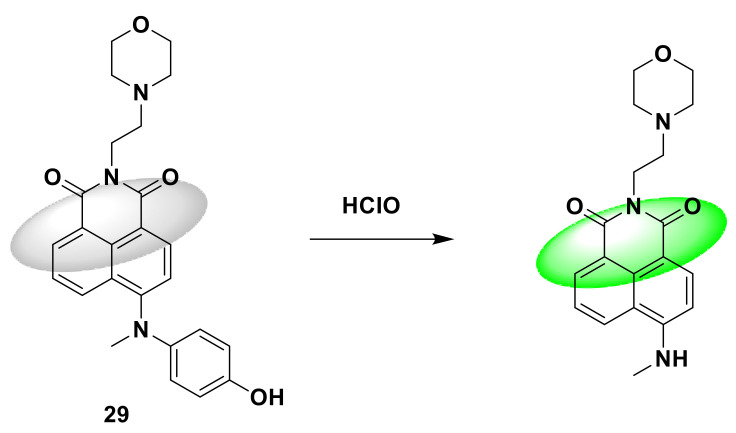
The reaction of probe 29 and HClO.

Wu et al. successfully designed a new naphthalimide-based two-photon fluorescent probe 29 [77], which consists of a lysosome targeting the morpholine group, the fluorescent recognition group naphthalimide and hypochlorite. The acid recognition group consists of aminophenol. The reaction with hypochlorous acid induces the oxidation of aminophenol, and the fluorescence increases by 136 times. The detection limit is 13 nM. Fluorescence enhancement is achieved by inhibiting twisted intramolecular charge transfer (TICT). This fluorescent probe shows high selectivity to hypochlorous acid and has a strong fluorescence response (Figure 29). After adding ROS such as H_2_O_2_, NO^2^^−^, ONOO^−^, NO^3^^−^, ^1^O_2_, O^2^^−^, and t-BuOOH, there is almost no fluorescence change; imaging experiments show that the probe can image endogenous lysosomal hypochlorous acid in living cells with a colocalization coefficient of up to 0.96. This provides an effective tool for studying the role of hypochlorous acid in biological research and medical diagnosis.

**Figure 30:**
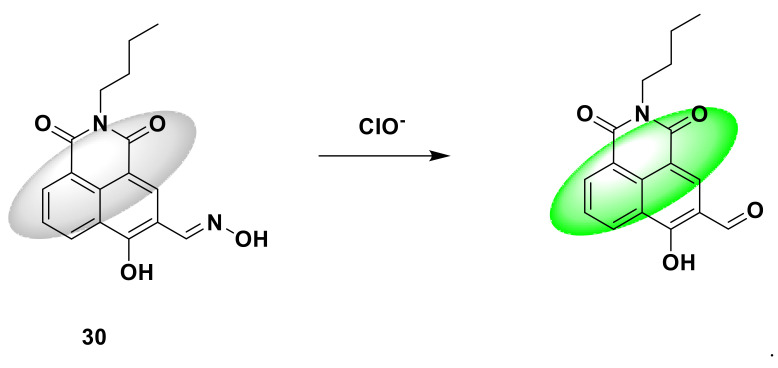
The reaction of probe 30 and ClO^−^.

A fluorescent probe 30 based on naphthalimide was designed and synthesized by Guo et al. [78]. The advantage of this probe is the short response time, reaching the maximum response in less than 3 s, and the large Stokes shift (75 nm). The probe containing C=N bond is non-fluorescent. After reacting with sodium hypochlorite solution, the probe is redoxed to remove the C=N bond in the naphthalimide, the fluorescence is significantly enhanced, and green fluorescence is produced (Figure 30). Its quantum yield increased from 0.006 to 0.389. The fluorescence intensity at 510 nm has a linear relationship with the concentration of NaClO in the range of 1–200 µM, and the detection limit is 2.88 nM. In this article, RAW264.7 macrophages were selected to verify the ability of the probe to image HClO/ClO^−^ in living cells. After incubating the probe (5 μM) with RAW264.7 macrophages for 20 min, there is almost no fluorescence in the cells. Another RAW264.7 macrophage was treated with PMA (100 ng/mL) for 2 h, and then incubated with the probe (5 μM) for 20 min, and strong green fluorescence was observed. These results demonstrate that the probe is cell-permeable and can be used to image endogenous hypochlorous acid in cells.

### 2.6. Others

**Figure 31:**
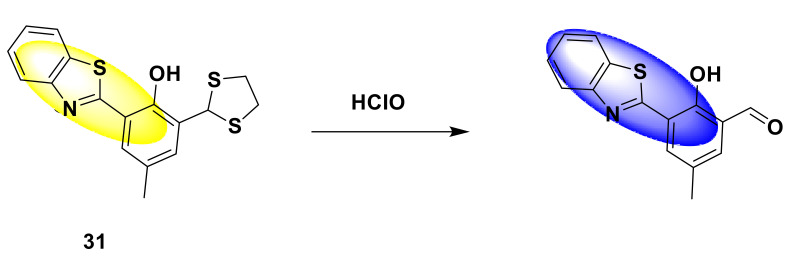
The reaction of probe 31 and HClO.

There are also many probes based on other fluorophores for the detection of ClO^−^. For instance, Chang et al. developed a benzothiazole-based hypochlorite ratio fluorescence sensor 31 with high sensitivity and selectivity [79]. The disulfide functional group in the probe reacts with hypochlorite. After adding the same amount of hypochlorite, the probe produces fluorescence from yellow to blue (Figure 31). In PBS buffer (10 mM, pH = 7.4), under excitation at 368 nm, the probe has a clear emission peak at 546 nm. With the addition of hypochlorite, a new fluorescence peak appeared at 464 nm, and the fluorescence peak appeared as a blue shift. The fluorescence intensity ratio (I_464_ nm/I_546_ nm) has a good linear relationship with ClO^−^ (0–10 equiv.). The detection limit is 8.9 nM. At the same time, when the pH is in the range of 6 to 9, it will not have a great impact on the probe. In cell imaging experiments, when the probe (10 μM) is incubated with HeLa cells, yellow fluorescence can be observed. Subsequent incubation with sodium hypochlorite showed blue fluorescence. After cells were treated with stimulating MPO (1.5 U per 100 mL) for 10 min and incubated with the probe for 10 min, blue fluorescence was observed, which means that MPO can stimulate the generation of endogenous secondary chlorate, which causes the fluorescence to change from yellow to blue. Therefore, the probe can be used for the detection of hypochlorite in living cells.

**Figure 32:**
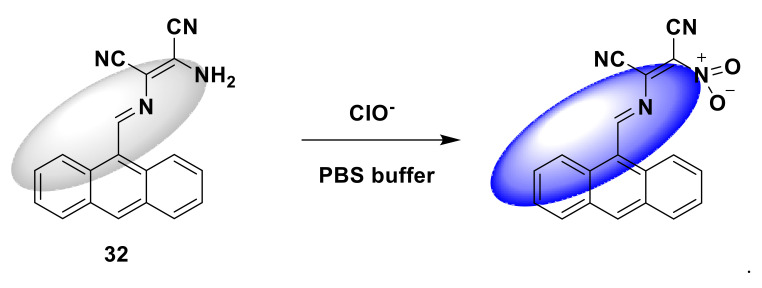
The reaction of probe 32 and CIO^−^.

In 2019, Wang et al. designed and synthesized an anthracene fluorophore-based hypochlorite detection turn-on fluorescent probe 32 [80]. In PBS buffer solution (10 mM, pH = 7.4, containing 1% DMF), after adding ClO^−^ (30 μM) to the probe (10 μM), it emits fluorescence signal (λex = 365 nm), and the fluorescence enhancement increases by 145 times. The detection limit is as low as 3.17 nM (Figure 32). The probe can effectively detect ClO^−^ in aqueous solution, with high selectivity, a fast response time, and a wide pH range. In addition, the probe can be used to detect endogenous ClO^−^ in RAW 264.7 macrophages and exogenous ClO^−^ in HeLa cells. This fluorescent probe will serve as a potential biological monitoring tool.

**Figure 33:**
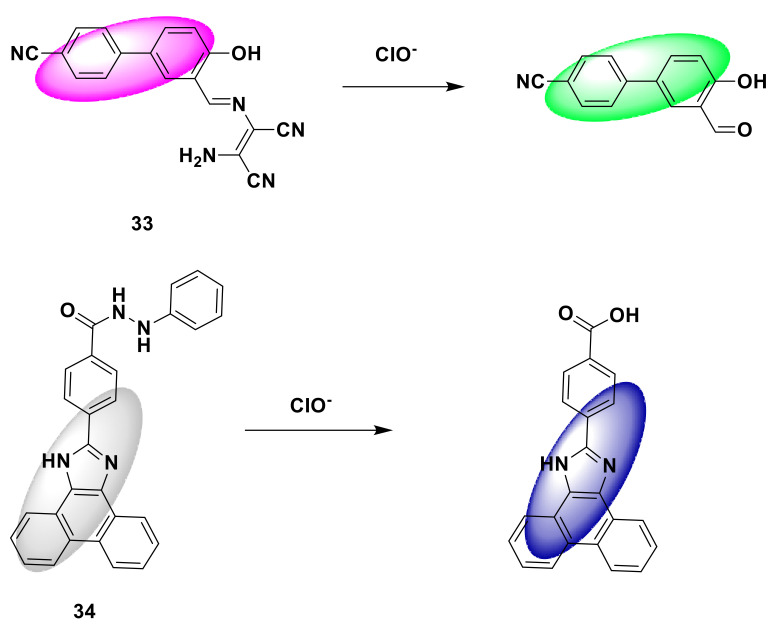

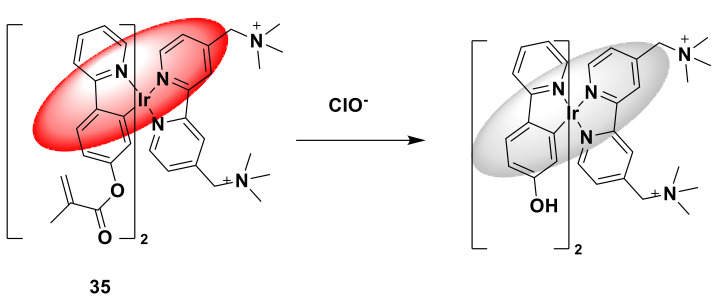
The reactions of probe 33, 34, 35, and ClO^−^.

In addition, Tang et al. reported a cyanobiphenyl-based hypochlorite ratio colorimetric fluorescent probe 33 [81]. After ClO^−^ was added to the probe solution, the hydrazone bond was induced to break, and the fluorescence spectrum (107 nm) occurred. A large blue shift caused the fluorescent color to change from red to green (Figure 33). Guo et al. synthesized a novel fluorescent probe 34 based on phenanthroline imidazole derivative [82]. The amide bond added to the ClO^−^ probe was oxidized and separated from the phenylhydrazine group. The fluorescence enhancement at 415 nm increased by about 20 times, and the detection limit was 0.58 mM (Figure 33). Cell imaging experiments show that the probe can detect ClO^−^ in living cells. Lu et al. synthesized a water-soluble fluorescent probe 35 based on cyclometalated iridium(III) complex [83]. The probe showed a strong fluorescence signal at 615 nm (Figure 33), while the fluorescence was quenched when ClO^−^ was added, and the detection limit was as low as 0.41 μM. In addition, the probe shows a fast response (<30 s) to ClO^−^, with high selectivity and sensitivity. In bioimaging, the probe has successfully detected ClO^−^ in live HepG2 cells.

## 3. Conclusions

In summary, this article reviews recent fluorescent probes based on different fluorophores for the detection of ClO^−^ in vivo and in vitro. Some of these probes can be used to monitor ClO^−^ in mitochondria, endoplasmic reticulum, and lysosomes. Probe design mechanisms include photoelectron transfer (PET), fluorescence resonance energy transfer (FRET), intramolecular charge transfer (ICT), etc. This article reviews its design strategy, sensing mechanism, optical performance, and biological application.

Although these probes exhibit excellent performance, such as photostability, short response time, high sensitivity, and high selectivity, most of the existing fluorescent probes used for HClO detection are poorly water-soluble, which is not suitable for living cells. Therefore, there is also an urgent need to discover new fluorescent probes that are highly sensitive and accurately detect complex environments.

## Data Availability

Not applicable.
